# Action of the Esculapian Club

**Published:** 1908-11

**Authors:** 


**Action of the Esculapian Club.**

Whereas, The hospital facilities in the city of Buffalo for the care and treatment of contagious diseases are inadequate and a menace to the health and welfare of the community; and

Whereas, We, the members of the Esculapian Club, have frequently been embarrassed in our treatment of contagious diseases and our patients have suffered through a lack of proper hospital accommodations: therefore

Be it *Resolved.* That the Esculapian Club commends the efforts now being made by the Buffalo Academy of Medicine and the Medical Society of the County of Erie for the establishment of a municipal contagious hospital, and that we heartily favor the establishment of such a hospital;

Be it *Resolved,* That copies of these resolutions be sent to both the above named societies, his Honor the Mayor, the President of the Board of Aldermen, the President of the Board of Councilmen, and the President of the Common Council.

Unanimously passed September 17, 1908.

George B. Stocker, M. D., Secretary.

The regular stated meeting of the Buffalo Academy of Medicine held in the Academy room of the Buffalo Public Library, Tuesday evening, October 13, was devoted exclusively to discussion of the subject of a Municipal Hospital for infectious and contagious diseases.

DISCUSSION.

Dr. Ullman, chairman of the committee, appointed some months since to investigate this subject, which has been acting in conjunction with a committee of fifteen from the Medical Society of the County of Erie presented an exhaustive report, from which it was only too apparent that Buffalo, as compared to other cities of its size and importance in this country and abroad is lamentably behind the times in its efforts, or rather lack of effort, to care for those suffering from these diseases.

The chief paper of the evening was presented by Dr. R. J. Wilson, superintendent of hospitals for infectious and contagious diseases of New York city. Dr. Wilson, aside from all humanitarian considerations, showed the serious economic loss suffered by the city, as well as the unfortunate victims of disease, where whole families and sometimes several families were unable to follow their usual avocations during several weeks of quarantine.

Dr. Gram, by special request, illustrated the tack-card system in use by the Buffalo Board of Health for the purpose of recording the location of various infectious diseases. The impression made upon his audience, especially by the cards representing deaths from scarlet fever and tuberculosis was most profound. One needs to see the impartial distribution among rich and poor, sanitary and unsanitary, to have brought home to him in a personal way, his relationship to these devastating and preventable diseases. He also read the following letter from Health Commissioner Wende. See letter p. 220.

The discussion of the reports and papers was opened by Dr. DeLancey Rochester, chairman of the sub-committee from the Medical Society of the County of Erie, which has been acting in conjunction with the committee from the Academy of Medicine. Dr. Rochester very briefly reinforced the arguments of the previous speakers in regard to the great necessity for a municipal hospital for the care of infectious and contagious diseases. He explained that it should be limited to certain diseases only, such as scarlet fever, diphtheria, measles and erysipelas. He wished it to be particularly understood that Dr. Wende in his letter did not intend to associate tuberculosis with the disease mentioned, but that there should be a separate institution; that while we are considering the necessity for the scientific care of other infectious diseases, we should not lose sight of the "Great White Plague"; that the city should provide adequate means for the treatment of its own tubercular patients. There is only Raybrook, crowded to the doors and with a waiting list of over two hundred, where the tuberculous poor of the state of New York can find accommodation. Erie County Hospital is also overcrowded, and always has many advanced cases, which exert a very seriously depressing effect on those of an incipient character. He emphasised the fact, brought out by Dr. Wilson, that a hospital for acute infectious diseases should be located near the center of population; that instead of being a danger to its neighborhood, the history of such institutions in New York and Boston prove that it tended to curtail the occurrence of these diseases in its immediate vicinity.

Col. Haffa, chairman of the board of aldermen, declared that he was fully convinced of the necessity for such an institution in the city of Buffalo. He quite agreed with Dr. Rochester that a tuberculosis hospital should be built. Dr. Gram's cards had been the most convincing evidence ever presented to him; and he trusted that they would be brought before the Board of Aldermen at the public hearing which would be given at 2.30 P.M., October 29.

Dr. J. G. Bonnar, chairman of the committee on contagious diseases of the Medical Society of the County of Erie, on behalf of his committee expressed his obligation to the various civic bodies who had manifested their interest in the subject by sending delegates to this meeting. He also raised an earnest plea for harmonious action on the part of all concerned in order to accomplish this great good in behalf of sufferers, who were in no way responsible for the great inconvenience and suffering they were compelled to bear, under the present existing conditions.

Alderman F. J. Eberle expressed himself as very heartily in accord with all that had been said on the subject.

Dr. Sigmund Goldberg, delegate from the East Buffalo Business Men's Association, said that they had had a two hours' discussion of the subject, and that it was the unanimous desire of the organisation of nearly one thousand members, that he should represent them before the Board of Aldermen, and expressed their opinion and most earnest desire that this necessity be consummated as early as possible.

Mr. Edward F. Dold, delegate from the Manufacturers' Club stated that they had a meeting last Tuesday, when it was unanimously decided to send a large committee to the board of aldermen on October 29 in behalf of this movement. They considered an institution of this sort a very great necessity.

Dr. Mary Denton assured the members of the Academy that the Federation of Women of Buffalo would give them their very hearty support, and that they might count on their presence in large numbers at the hearing on October 29.

On motion of Dr. Rochester, the report of the commission was unanimously adopted, and it was continued with power to act further in the establishment of such an institution.

